# Uncorrected amteropia among children hospitalized for headache evaluation: a clinical descriptive study

**DOI:** 10.1186/1471-2431-14-241

**Published:** 2014-09-29

**Authors:** Gad Dotan, Chaim Stolovitch, Elad Moisseiev, Shlomi Cohen, Anat Kesler

**Affiliations:** Department of Ophthalmology, Sourasky Tel Aviv Medical Center, 6 Weizmann Street, Tel Aviv, 64239 Israel; Sackler School of Medicine, Tel Aviv University, Tel Aviv, Israel; Pediatric Gastroenterology Unit, ‘Dana-Dwek’ Children’s Hospital, Sourasky Tel Aviv Medical Center, Tel Aviv, Israel

**Keywords:** Headache, Asthenopia, Amteropia, Refractive error

## Abstract

**Background:**

Headache is a common complaint in children occasionally requiring hospital admission. The purposes of the present study were to analyze the prevalence of uncorrected ametropia in children with headache admitted to the hospital, and evaluate the importance of refraction assessment as part of their evaluation.

**Methods:**

A retrospective review of children admitted to the Tel Aviv Medical Center for headache evaluation from December 2008 to March 2013, in whom the only abnormality found was an uncorrected refractive error.

**Results:**

During the study period 917 children with headache were hospitalized for evaluation and 16 (1.7%) of them (9 boys, mean age 12 years, range 8–18 years) were found to have an uncorrected ametropia. Average headache duration was 4 months (range, 1 week to 1 year) and mean follow-up was 15 months (range, 1 month to 3 years). Twelve (75%) children had brain imaging and 4 children (25%) had a lumbar puncture before their refractive abnormality was identified. Anisometropia and myopia were the most common refractive errors encountered (n = 10 each), followed by hyperopia (n = 6) and astigmatism (n = 3). Despite having uncorrected refractive errors most children (n = 10) did not complain of any visual difficulty. All children were given proper refractive correction and 14 of them reported complete headache resolution on re-examination one month later.

**Conclusions:**

Uncorrected ametropia is a possible cause of headache among hospitalized children. Therefore, complete ophthalmic evaluation, which includes proper refraction assessment, is important as it can identify a treatable headache etiology. Children without visual difficulty should be equally evaluated, as many children with headache and uncorrected amteropia do not have vision complaints.

## Background

It is well recognized that certain eye conditions such as acute glaucoma, posterior scleritis or optic neuritis may be associated with eye pain and/or headache [1]; however there is much less certainty that uncorrected refractive errors are a possible source of headache. The diagnostic criteria for headache associated with refractive errors defined by the International Headache Society are: 1. Uncorrected or miscorrected refractive errors such as hyperopia or astigmatism, 2. Mild headache in the frontal region and in the eyes themselves, 3. Pain absent on awakening and aggravated by prolonged visual tasks at the distance or angle where vision is impaired. Disappearance of the headache following successful treatment of the underlying refractive disorder is another important diagnostic criterion [2].

Several authors [3–7] reported on possible association between refractive errors and headache in both children and adults; whereas others [8–11] reported on the contrary believing that the finding of an abnormal refractive error in an individual with headache is purely coincidental, based on the high prevalence of both conditions in the general population, which does not imply a causal relationship between them.

The purposes of the present study were to analyze the prevalence of uncorrected ametropia in children with headache admitted to the hospital, and evaluate the importance of refraction assessment as part of their evaluation.

## Methods

The study was approved by the Tel Aviv Medical Center institutional review board and was fully compliant with the principles of the Declaration of Helsinki.

A retrospective analysis was conducted of the medical records of children 8 to 18 years old with headache admitted to our medical center between December 2008 and March 2013 in whom the only abnormality found was uncorrected ametropia. None of the children included in this study had any systemic or ocular diseases known to be associated with eye-strain or headache such as glaucoma, optic neuritis or scleritis, or a known refractive error necessitating the use of spectacles or contact lenses. Following a normal neurological examination, which revealed no underlying headache etiology all children underwent a complete ophthalmological evaluation by a pediatric neuro-ophthalmologist (GD) and were asked about difficulty with distance and near visual tasks before examination was initiated. In all children assessment of visual acuity, pupillary reaction, ocular motility, ocular misalignment, slit lamp biomicroscopy, dilated funduscopy and cycloplegic refraction were performed. Visual acuity was measured using a Snellen chart and was converted to the logarithm of the minimum angle of resolution (logMAR) for statistical analysis. Cycloplegic refraction was performed by retinoscopy following administration of two cycles of cyclopentolate 1% in each eye, 5 minute apart.

All children included in this study had uncorrected amteropia, which was defined according to the criteria reported by Akinci et al. [3]: myopia (spherical equivalent (SE) refraction of at least -0.50 diopters), hyperopia (SE of at least +2.00 diopters), astigmatism (a cylinder of at least 1.00 diopters), or anisometropia (SE difference of 1.00 diopter or more between the two eyes). None of the children had any other ophthalmological abnormality known to cause headache, including: convergence or accommodation insufficiency, heterophoria, heterotropia, or pseudotumor cerebri. All children were given proper refractive correction and were re-examined one month later.

Data were analyzed using Minitab release 14 software (Minitab Inc., State College, PA). Descriptive statistics are provided and for inference testing statistical significance was defined at a level of P < .05. Visual acuity at presentation and at follow-up was compared using a paired t-test.

## Results

During the study period 917 children with headache were hospitalized to the Tel Aviv medical center and in 16 (1.7%) of them (9 boys, mean age 12 years; range 8 to 18 years) the only abnormality found was an uncorrected ametropia. Average headache duration was 4 months (range, 1 week to 1 year) and mean follow-up was 15 months (range, 1 month to 3 years). Twelve (75%) children had a normal brain imaging prior to their ophthalmic examination. Five children had brain computerized tomography, five children had brain magnetic resonance imaging, and two children had both. Four (25%) children had a lumbar puncture as well, which demonstrated a normal cerebrospinal fluid opening pressure (16 – 21 cmH_2_0) with normal fluid constituents in all cases.

Although all 16 children had uncorrected ametropia (Table 1), 10 of them did not report having any visual difficulty. Types of refractive errors found were: anisometropia (n = 10), myopia (n = 10), hyperopia (n = 6), and astigmatism (n = 3). One month following prescription of optical correction 14 children had complete resolution of headache complaints. Best-corrected visual acuity at follow-up (0.01 ± 0.04) was significantly improved compared to presentation (0.25 ± 0.22, P < 0.001).

**Table 1:** **Refractive errors encountered in children with headache and visual acuities before and after optical correction**

Patient	Cycloplegic refraction RE (diopters)	Cycloplegic refraction LE (diopters)	Glassed prescribed RE (diopters)	Glasses prescribed LE (diopters)	Initial logMAR RE	Initial logMAR LE	Final logMAR RE	Final logMAR LE
1	-1.00 - 1.25 × 10	-0.50 - 1.25 × 170	-1.00 – 1.25 × 10	-0.50 – 1.25 × 170	0.6	0.2	0.0	0.0
2	-2.00 - 0.25 × 180	-2.00 - 0.25 × 180	-2.00 - 0.25 × 180	-2.00 - 0.25 × 180	1.0	1.0	0.0	0.0
3	-0.75 - 0.50 × 180	-0.75 – 0.50 × 180	-0.75 - 0.50 × 180	-0.75 – 0.50 × 180	0.3	0.2	0.0	0.0
4	+3.00 – 0.25 × 180	+2.00 – 0.25 × 180	+1.50 – 0.25 × 180	+0.50 – 0.25 × 180	0.2	0.0	0.1	0.0
5	0.00 - 3.50 × 130	+0.25	0.00 - 3.50 × 130	+0.25	0.3	0.0	0.0	0.0
6	-1.50	-1.75	-1.50	-1.75	1.0	0.7	0.0	0.0
7	+1.50 – 2.00 × 180	+2.00 – 2.25 × 170	0.00 – 2.00 × 180	+0.50 – 2.25 × 170	0.3	0.3	0.0	0.0
8	-1.00	-1.00	-1.00	-1.00	0.2	0.2	0.0	0.0
9	+4.00 – 0.75 × 180	+2.25 - 0.25 × 180	+2.50 - 0.75 × 180	+0.75 - 0.25 × 180	0.3	0.0	0.1	0.0
10	-1.00 – 0.75 × 90	-0.25 – 0.50 × 90	-1.00 – 0.75 × 90	-0.25 – 0.50 × 90	0.1	0.1	0.0	0.0
11	-1.00 – 0.25 × 90	-1.25 – 0.25 × 90	-1.00 – 0.25 × 90	-1.25 – 0.25 × 90	0.2	0.4	0.0	0.0
12	+0.50	+2.75	0.00	+2.25	0.1	0.3	0.0	0.2
13	+0.25	+3.50 – 0.50 × 180	0.00	+3.25 – 0.50 × 180	0.0	0.4	0.0	0.0
14	+1.25	+2.50	0.00	+1.25	0.0	0.0	0.0	0.0
15	-2.00	-1.00	-2.00	-1.00	0.4	0.2	0.0	0.0
16	+3.50 – 0.25 × 180	+4.75 – 0.50 × 180	+2.00 – 0.25 × 180	+3.25 – 0.50 × 180	0.2	0.3	0.0	0.1

RE = Right eye, LE = Left eye. LogMAR = logarithm of the minimum angle of resolution.

## Discussion

We describe 16 children who had headache requiring hospital admission, in whom the only abnormality found was the presence of uncorrected ametropia. Only a minority of these children reported having visual difficulties. One month following prescription of proper refractive correction there was resolution of headache complaints in most children.

Headache is a frequent pediatric complaint [6]. Hendricks et al. [6] reported that 70% of healthy children between the ages of 11 to 13 years had at least one headache episode during a period of 1 year and that 15% of them classified their headache as severe by burden or duration. Although there is a strong popular belief amongst physicians and parents that visual problems can be a cause of headache there is still a controversy in the literature whether such an association truly exists and uncertainty regarding the causal mechanism. Different types of refractive errors have been related to headache complaints. Hyperopia was found to be a possible cause of headache in the studies of Gil-Gouveia et al. [4] and Ip et al. [7] by causing painful contracture of the ciliary muscle evoked by sustained accommodation. Myopia can lead to headache from squinting of the forehead and eyelids in order to narrow the palpebral fissure, achieving a pinhole effect and improving vision [4]. Anisometropia and astigmatism were reported as an etiology of headache in the study of Akinci et al. by a mechanism, which is not fully understood, but is probably related to visual blur [3]. In our study the most frequently encountered refractive errors among children with headache related to uncorrected ametropia were anisometropia and myopia (both were present in 63% of children) followed by hyperopia (38% of children) and astigmatism (19% of children).

Akinci et al. [3] found a high prevalence of uncorrected and miscorrected refractive errors among children 8 to 18 years old with headache in whom an evaluation by a pediatric neurologist, otolaryngologist, and brain imaging did not reveal any pathology; however, they did not report on headache status following optical correction. In our study we have similarly identified 16 children with headache and uncorrected refractive errors in whom prior evaluation did not identify headache etiology. Gil-Gouveia et al. [4] found that headache was more common in individuals with uncorrected refractive errors compared to matched controls without this refractive abnormality. Seventy three percent of individuals had improvement in their complaints with optical correction, including 38% who had complete headache resolution. In our study, we have similarly found that proper optical correction can result in headache elimination. Miscorrection of refractive errors or unnecessary spectacle use can be a source for problem as well. In the study of Robaei et al. [5] children wearing glasses without having a significant refractive error in either eye were more likely to have eye-strain or headache complaints compared to all other children. Despite the fact that our study included only children with uncorrected refractive errors, it is clear that children with headache who are wearing glasses or contact lenses should be similarly evaluated for wearing the proper correction.

Contrary to the reports mentioned above, other studies found no association between refractive errors and headache. Korczyn et al. [8] found no difference in the frequency of headache in individuals with or without refractive errors, Waters [9] reported no difference in the visual acuity of individuals with or without headache, and Hedges [10] expressed his disbelief that refractive errors are an important cause for headache. These authors attributed the relief of headache following correction of a refractive error to a placebo treatment effect or a functional etiology.

According to Linet et al. [12] an ophthalmologist is the third most common practitioner consulted for headache. Ip et al. [7] reported that only a minority of 6-years-old children referred for ophthalmological evaluation due to headache or eye-strain complaints had uncorrected refractive errors or an identifiable ocular abnormality and recommended primary care givers to reconsider the need for an ophthalmological evaluation in children with headache complaints since it is often normal and has a low yield for revealing a treatable eye pathology. Even though we have found uncorrected ametropia in only 1.7% of children admitted to the hospital due to headache complaints we disagree with their conclusion. A complete eye examination, which includes assessment of refraction may identify a treatable cause of headache, reduce anxiety of existence of more serious pathologies and occasionally prevent the need for further investigations.

Our study has several limitations, which should be taken into consideration when interpreting its results. First, this was a retrospective record review and is subject to inherent limitations of such a design, mainly the reliance upon the completeness and accuracy of previous medical records. Second, we did not routinely complete a headache questionnaire and thus it is impossible for us to better characterize the type of headache complaints of children with uncorrected ametropia. Third, not all children with headache hospitalized during the study period underwent a complete eye examination with refraction assessment; therefore it is possible that the prevalence of uncorrected amteropia in children admitted with headache is even higher than reported in this study. Also, our study did not include a control group; therefore we cannot determine whether the prevalence of uncorrected ametropia found in children with headache is different than in children admitted for other reasons. However, despite its limitations our study provides important clinical information as we have found that uncorrected ametropia is a possible etiology of headache in admitted children who may not express any visual difficulty. In addition we found that optical correction often results in resolution of headache complaints. Larger, prospective case–control studies, employing headache questionnaires are still needed to better establish the association of uncorrected amteropia and headache.

## Conclusions

Uncorrected ametropia is a possible cause of headache among hospitalized children. Therefore, complete ophthalmic evaluation, which includes proper refraction assessment, is important as it can identify a treatable headache etiology. Children without visual difficulty should be equally evaluated, as many children with headache attributed to uncorrected amteropia do not report vision complaints.

## Ethics

This study was approved by the Helsinki committee of the Tel Aviv Medical Center, study no. 0559-12-TLV.
